# Drainage morphometric analysis of the Nagavathi watershed, Cauvery river basin in Dharmapuri district, Tamil Nadu, India using SRTM data and GIS

**DOI:** 10.1016/j.dib.2018.07.016

**Published:** 2018-07-19

**Authors:** R. Kannan, S. Venkateswaran, M. Vijay Prabhu, K. Sankar

**Affiliations:** a Periyar University, Salem, Tamil Nadu, India; b Tamil University, India

**Keywords:** Arc GIS, Drainage density, Rainwater harvesting, SRTM data and Stream Order

## Abstract

A drainage morphometric analysis of Nagavathi watershed in Dharmapuri district has been chosen for the present study. Geospatial tools, such as remote sensing and GIS, are utilized for the extraction of watershed and its drainage networks. The Shuttle Radar Topographic Mission (SRTM) data have been used for drainage morphometric analysis and evaluating various morphometric parameters Linear aspect, Aerial aspect Relief aspect. The morphometric parameters of Nagavathi watershed have been analyzed and evaluated by pioneer methods, such as Horton and Strahler. The bifurcation ratio varies from 0.8 to 43.1. The elongation ratio of Microwatersheds varies from 0.13 to 0.43, indicates Microwatersheds fall under elongated pattern. This study would help the local people to utilize the resources for planning rainwater harvesting and watershed management.

**Specification Table**

**Table:** 

**Subject area**	Hydrogeology
**More specific subject area**	Watershed Management
**Type of data**	Table and Figure
**How data was Acquired**	Data format Raw, Digitized
**Experimental factors**	The mentioned parameters above, in the abstract section, were derived the formula in publishing papers
**Experimental features**	Determination of morphological analysis that constitute the Nagavathi watershed
**Data source location**	It lies between latitudes 11°45’N to 12°15’ N and 77°30’ E to 78°30 E longitudes covering an area of about 482 Km^2^
**Data accessibility**	All the data are in this data article

**Value of the data**

• The data utilize the resources for planning rainwater harvesting and watershed management.
• The data set can be used for educational purposes, and for future research in watershed Morphometric studies.
• The data show the relationship occurring between the surface and subsurface of the groundwater.
• The data could be used in management groundwater potential.

## 1 Data

The data contains morphometric analysis of the Nagavathi watershed in Dharmapuri district of Tamil Nadu. The data are composed of Shuttle Radar Topographic Mission Digital Elevation Model (SRTM - DEM) data. Derived from mathematical equations Table 1. Results of the watershed morphometric analysis are presented in Table 2.

**Table 1:** Morphometric parameters and their mathematical expressions.

**S.No**	**Parameter**	**Formula**
**Linear aspect**		
1.	Area (A)	Area of the watershed
2.	Perimeter (P)	The perimeter is the total length of the watershed boundary.
3.	Length (Lb)	Maximum length of the watershed
4.	Stream Order (Nu)	Hierarchical rank
5.	Stream Length(Lu)	Length of the stream
6.	Stream length ratio (Rl)	Rl=Lu/Lu−1
7.	Mean Stream Length Ratio (Lsm)	Lsm=Lu/Nu
8.	Bifurcation ratio (Rb)	Rb=Nu/*N*(u+1)

**Areal aspect**		
9.	Drainage density (Dd)	Dd=∑Lu/A
10.	Stream frequency (Fs)	Fs=∑Nu/A
11.	Texture Ratio	*T*=Nu/*P*
12.	Elongation ratio (Re)	Re=1.128√*A*/*L*
13.	Form factor (Ff)	Ff=*A*/Lb^2^
14.	Circularity index (Rc)	Rc=4*πA*/*P*^2^
15.	Length of overflow (Lg)	Lg=1/2/2*d*
16.	Constant of Channel maintenance (Ccm)	*C*=1/*Dd*
17.	Drainage texture (T)	*T*=*Dd*×Fs
18.	Compactness coefficient (Cc)	Cc+0.282*P*/√*A*^0.5^

**Relief aspect**		
19.	Basin relief (R)	*R*=*H*−*h*
20.	Relief ratio (Rr)	Rr=*R*/*L*
21.	Ruggedness number (*Rn*)	*Rn*=*R*×*Dd*
22.	Gradient ratio (*G*r)	*G*r=(*H*-*h*)/*L*
23.	Melton ruggedness ratio (MRn)	MRn=(*H*-*h*)/*A*^0.5^
24.	Slope (Sb)	Sb=*H*−*h*/*L*
25.	Relative relief (Rhp)	Rhp=*H*/*P*×100
26.	Shape Factor (Rf)	Rf=*Lb*^2^/*A*
27.	Leminscate(K)	*K*=*Lb*^2^/4×*A*

**Table 2:** Linear, areal and relief aspects of Nagavathi watershed.

**S. No**		**Parameter**		**MWS01**	**MWS02**		**MWS03**	**MWS04**		**MWS05**		**MWS06**		**MWS07**		**MWS08**
**Linear aspect**																
1.	Area (A)			72.18	58.76		33.35		42.14		43.43	82.79		32.26		116.45
2.	Perimeter (P)			42.21	36.27		27.83		35.45		43.43	46.39		24.39		66.03
3.	Micro Watershed Length (LW)			7.26	7.56		6.23		3.16		10.71	7.04		6.16		21.55
4.	No. of Stream Order (Nu)			67	53		38		43		47	78		36		151
5.	Stream Length (Lu) km			78.86	61.49		40.85		44.64		54.27	75.71		40.51		128.28
6.	Stream Length Ratio (Rl)		II/I	0.63	0.45		0.43		0.67		0.66	0.47		0.36		0.96
			III/II	0.11	0.13		0.20		0.22		0.27	0.11		0.17		0.24
			IV/III	0.08	0.14		0.11		0.26		0.13	0.25		0.19		0.13
			V/IV	0.00	0.00		1.67		0.00		0.49	0.00		0.25		20.00
7.	Mean Stream Length Ratio (Lsm)			4.5	4.1		43.1		4.8		1.8	4.2		3.2		0.8
8.	Bifurcation Ratio (Rb)		I/II	2.9	3.6		3.9		3.3		3.0	3.7		4.6		2.3
			II/III	8.3	8.0		5.5		5.0		5.7	7.7		4.5		9.7
			III/IV	4.0	3.0		3.0		4.0		4.0	4.0		3.0		4.5
			IV/V	0.0	0.0		0.0		0.0		2.0	0.0		2.0		3.0

**Areal aspect**																
9.	Drainage density (Dd)			1.1	1.0	0.5			0.5		0.2		0.9		1.7	0.1
10.	Stream frequency (Fs)			0.93	0.90	1.14			1.02		1.08		0.94		1.12	1.30
11.	Texture Ratio			0.97	0.99	0.93			0.76		0.64		1.16		1.03	1.21
12.	Elongation ratio (Re)			0.13	0.14	0.27			0.25		0.38		0.13		0.15	0.43
13.	Form factor (Ff)			0.01	0.02	0.10			0.10		0.55		0.01		0.01	2.52
14.	Circularity index (Rc)			0.51	0.56	0.54			0.42		0.29		0.48		0.68	0.34
15.	Length of overflow (Lg)			0.46	0.48	0.93			1.02		2.44		0.55		0.29	8.56
16.	Constant of Channel Maintenance (Ccm)			0.92	0.96	1.86			2.05		4.88		1.11		0.58	17.13
17	Drainage texture (T)			1.6	1.5	1.4			1.2		1.1		1.7		1.5	2.3
18.	Compactness coefficient (Cc)			1.40	1.33	1.36			1.54		1.86		1.44		1.21	1.73

**Relief aspect**																
19.	Basin relief (R)			500	517	500			892		515		537		540	510
20.	Relief ratio (Rr)			6.4	8.4	27.9			43.3		57.9		7.2		9.8	75.0
21.	Ruggedness number (*Rn*)			545.16	539.35	268.52			436.05		105.54		485.17		924.33	29.78
22.	Gradient ratio (*G*r)			6.4	8.4	27.9			43.3		57.9		7.2		9.8	75.0
23.	Melton Ruggedness ratio (MRn)			58.9	67.4	86.6			137.4		78.1		59.0		95.1	47.3
24.	Basin Slope (Sb)			18.7	20.8	21.3			16.8		3.9		0.5		0.6	3.6
25.	Relative relief (Rhp)			11.85	14.25	17.97			25.16		11.86		11.58		22.14	7.72
26.	Shape Factor (Rf)			85.81	63.95	9.62			10.07		1.82		67.58		94.52	0.40
27.	Leminscate(K)			21.45	15.99	2.40			2.52		0.46		16.90		23.63	0.10

The quantitative morphometric analysis was carried out in eight Micro watersheds of Nagavathi watershed using GIS technique for determining (a) Linear aspects like Stream number, Stream order, Stream length, Mean stream length, Stream length ratio, Bifurcation ratio, (b) Aerial aspects like Drainage density, Stream frequency, Texture Ratio, Elongation ratio, Form factor, Circularity index, Length of overflow, Constant of Channel maintenance, Drainage texture, Compactness coefficient and (c) Relief aspects like Basin relief, Relief ratio, Ruggedness number, Gradient ratio, Melton ruggedness ratio, Slope, relative relief, Shape Factor and Leminscate.

## 2 Study areas

**Fig. 1:**
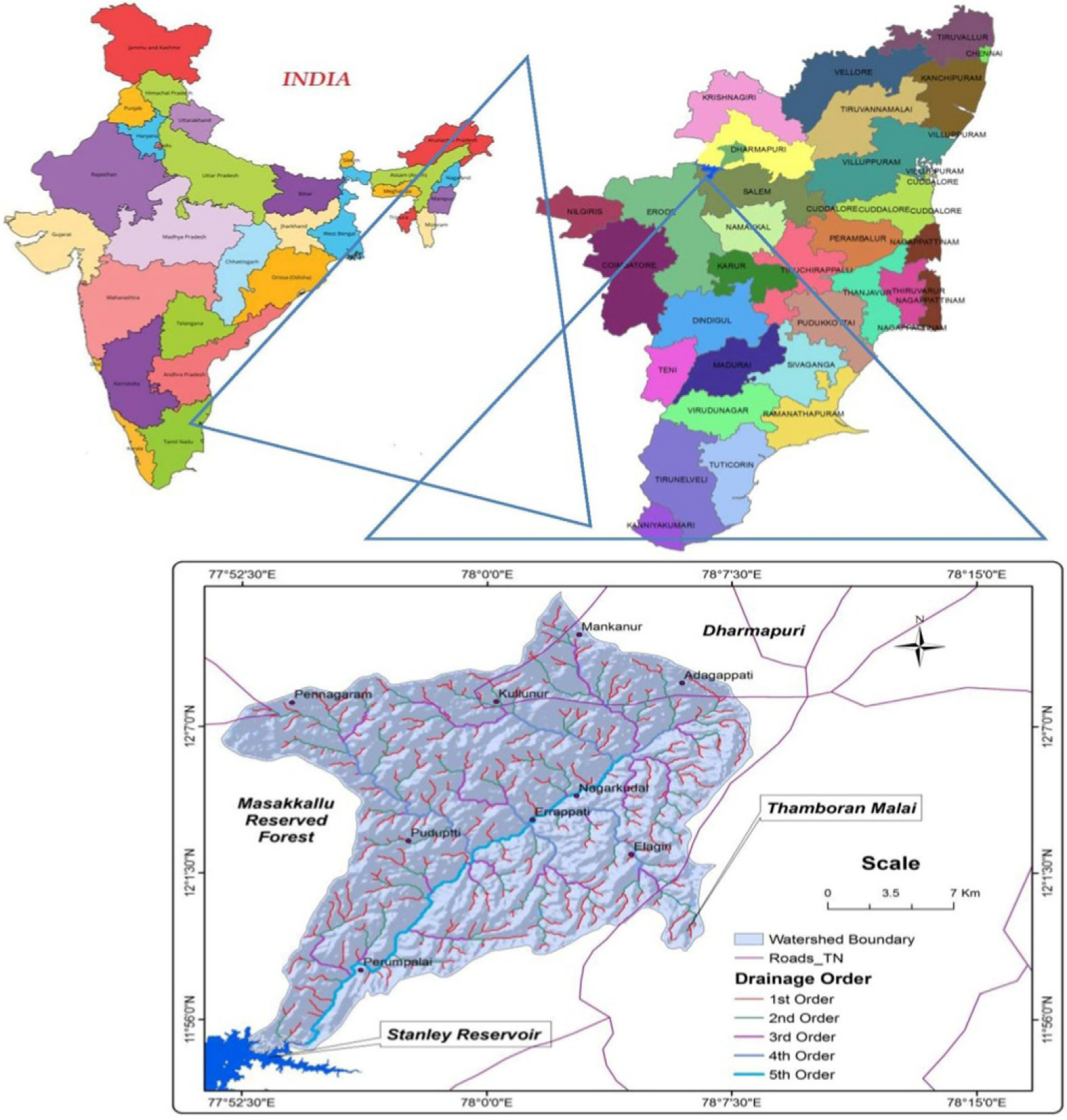
Location map of the Nagavathi.

Nagavathi watershed is located in part of Dharmapuri district of Tamil Nadu. It lies between latitudes 11°45′N to 12°15′ N and 77°30′ E to 78°30 E longitudes covering an area of about 482 Km^2^ (Fig. 1). The climate of the Dharmapuri district is generally warm. The hottest period of the year is generally from the months of March to May, the highest temperature going up to 38 °C in April. The Climate becomes cool in December and continuous so up to February, touching a minimum of 17 °C in January. The Soil type ranges from black to mixed loam, Red sandy soils and black and loam soil are found in the watershed. Generally the soil is low in nitrogen and phosphate content. Geology of area is underlined by a wide range of igneous and metamorphic rocks. The geological formations of the study area are under Archean group representing Champion gneiss, charnockite, syenite, pink pegmatite and pyroxene granulite. The charnockites and associated pink migmatities mostly occupy the study area. Champion gneiss is dominant rock in the study area. It is highly pink migmatized at many places and show deep weathering.

## 3 Methods and materials

**Fig. 2:**
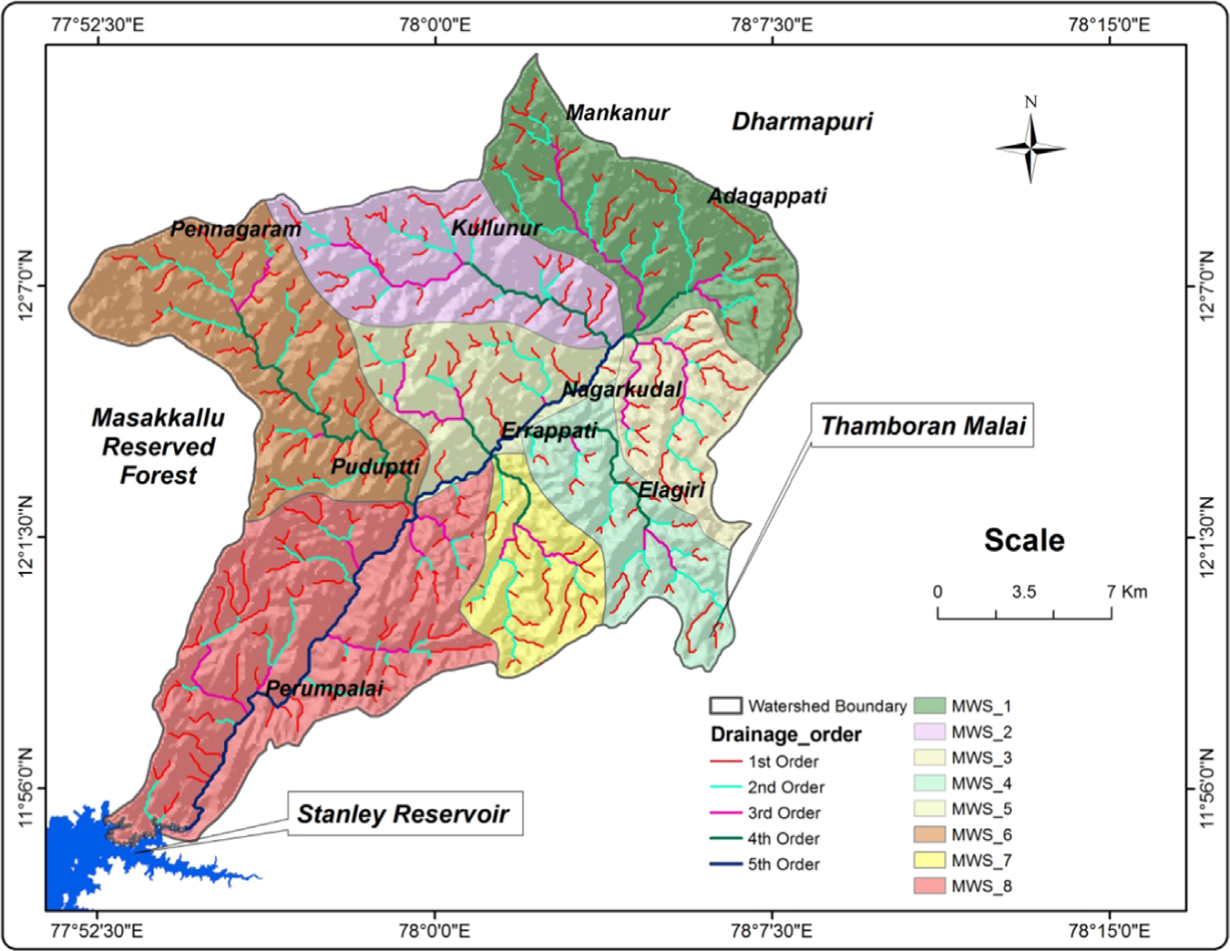
Drainage and microwatershed – map.

Geological Survey of India (GIS) topographical maps of 1:50,000 scales were used to prepare Base maps and watershed Drainage maps (Fig. 2) of Nagavathi watershed of Cauvery river basin, Tamil Nadu. Stream network for the above watershed are traced and scanned. The scanned stream network map was geo referenced and converted into digital format using Arc GIS 9.3 version GIS software. The data used in this study include 30 m resolution Digital Elevation Model (DEM) of the basin extracted from the Shuttle Radar Topographic Mission (SRTM) downloaded from the US Geological Survey Website. Quantitative morphometric analysis was carried out for eight in the watershed as mentioned above for linear aspects, areal aspects and relief aspects. The analysis was carried out using Arc GIS software. The drainage network generated was then analysed using [1], [2], [3], [4], [5], [6], [7], etc. for various parameters.

## 4 Stream direction

**Fig. 3:**
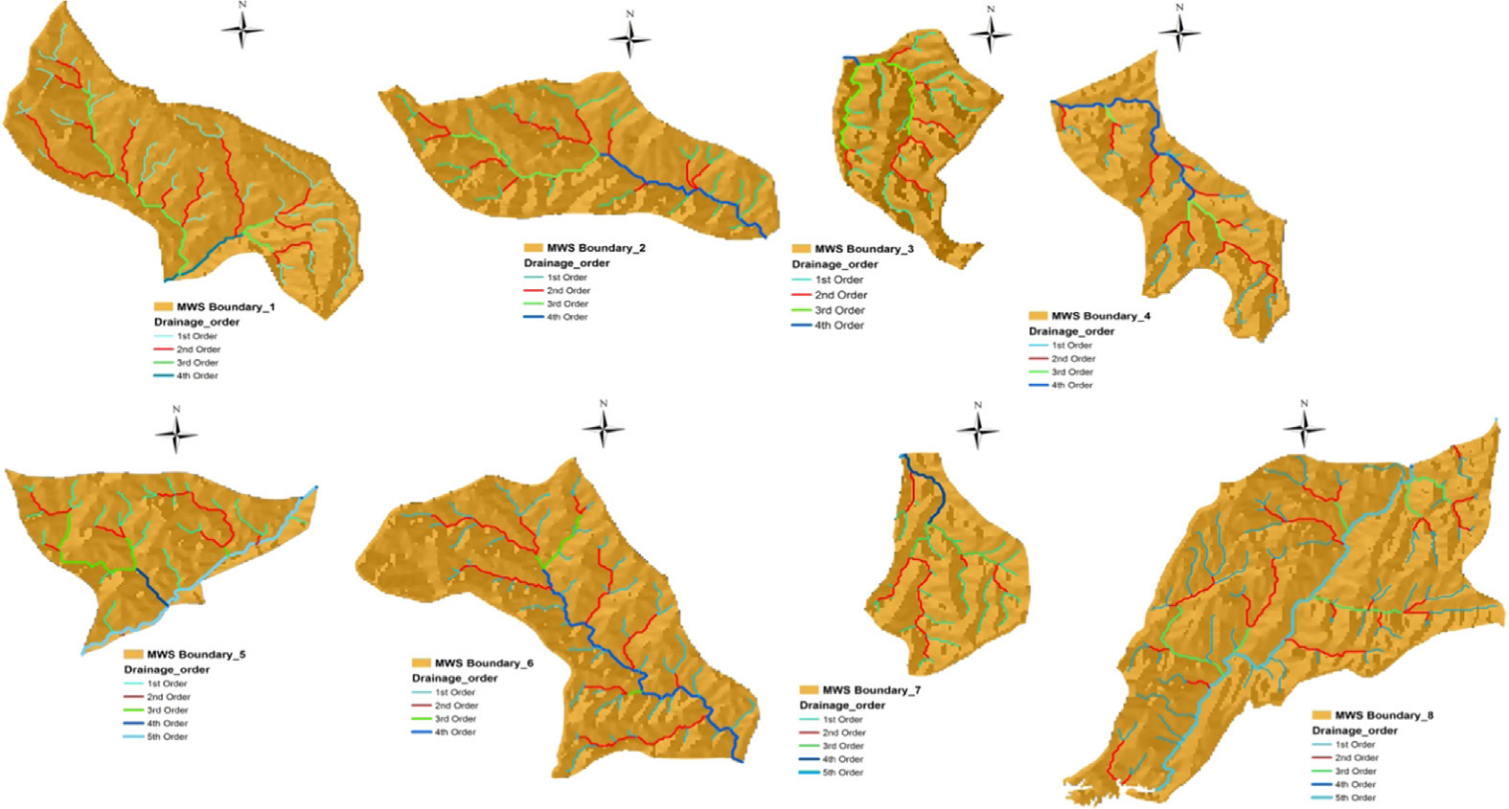
Micro watershed–map.

**Fig. 4:**
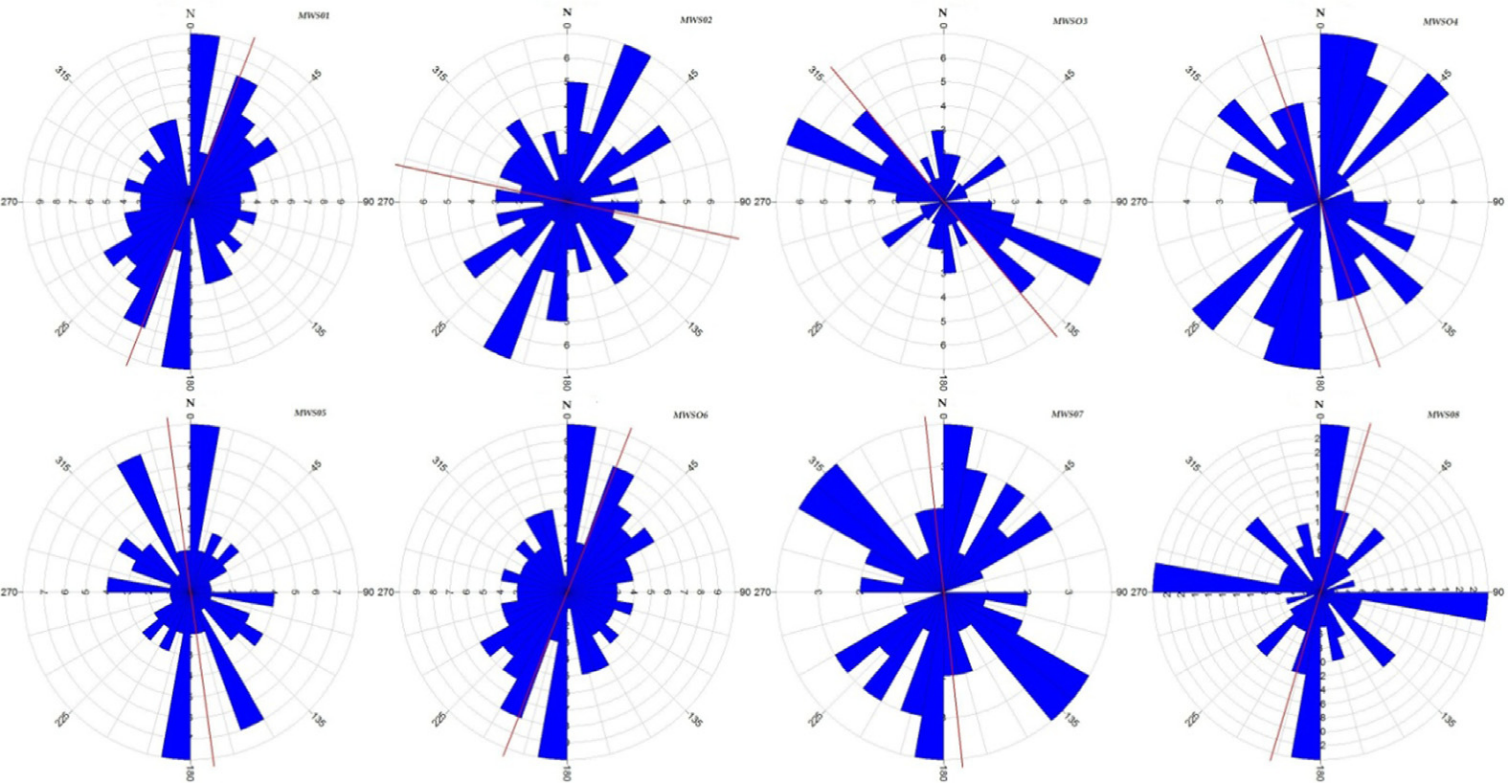
Rose diagrams show the geometry of the streams direction and length.

The stream direction has been computed to understand the surface flowing pattern for the surface water development. The length and its direction of each drainage line have been calculated in GIS environment and the values are plotted in Rockworks software for each all micro watershed, presented in Figs. 3 and 4. The stream tributary directions and the local tectonic regime, the stream channels of the Nagavathi micro watershed were grouped according to their order (1–5) and eight rose diagrams were created for each watershed. The major and minor lineament, that is upstream and downstream sections of the watershed, respectively. The watershed in northeast-southwest direction with micro watersheds like MWS01, MWS06 and MWS08. The lineament crosses the watershed in a southwest-northeast direction and MWS02, MWS03, MWS04, MWS05, and MWS07 in the Microwatershed. In the all micro watershed the dominant direction for maximum streams order is NE-SW and all direction in the micro watershed.
