# Supplementary material for: Drainage morphometric analysis of the Nagavathi watershed, Cauvery river basin in Dharmapuri district, Tamil Nadu, India using SRTM data and GIS
Source: Data Brief. 2018 Jul 19;19:2420–6. doi: 10.1016/j.dib.2018.07.016 (PMC6141492; doi:10.1016/j.dib.2018.07.016)
Supplement: Supplementary file 1 — Supplementary material [file mmc1.doc]

**CONFLICT OF INTEREST FORM DATA IN BRIEF ELSEVIER**

Manuscript title: **Drainage Morphometric Analysis of the Nagavathi Watershed, Cauvery River basin in Dharmapuri District, Tamil Nadu, India Using SRTM Data and GIS**

The authors whose names are listed immediately below certify that they have NO affiliations with or involvement in any organization or entity with any financial interest (such as honoraria; educational grants; participation in speakers’ bureaus; membership, employment, consultancies, stock ownership, or other equity interest; and expert testimony or patent-licensing arrangements), or non-financial interest (such as personal or professional relationships, affiliations, knowledge or beliefs) in the subject matter or materials discussed in this manuscript.

Author names:

**by** **Kannan R, Venkateswaran S, Vijay Prabhu M and Sankar K**
